# Reliability of Chest Wall Mobility and Its Correlation with Lung Functions in Healthy Nonsmokers, Healthy Smokers, and Patients with COPD

**DOI:** 10.1155/2019/5175949

**Published:** 2019-02-25

**Authors:** Ravi S. Reddy, Khalid A. Alahmari, Paul S. Silvian, Irshad A. Ahmad, Venkata Nagaraj Kakarparthi, Kanagaraj Rengaramanujam

**Affiliations:** Department of Medical Rehabilitation, King Khalid University, Abha 61421, Saudi Arabia

## Abstract

Chest wall circumference measurements are common evaluation methods in clinical settings by therapists in order to obtain chest wall mobility. Previous published results have been conflicting, and there is a lot of variability in the method of testing, which needs testing in different conditions. Seventy subjects (25 healthy nonsmokers, 25 healthy smokers, and 20 COPD) aged between 18 and 70 years participated in the study. Upper and lower chest expansion (CE) measurements (2 levels) are performed with cloth inch tape. Intrarater (between day) and interrater (within-day) reliability of CE measurements was evaluated by two examiners. Lung function parameters, forced expiratory volume in first second (FEV1), forced vital capacity (FVC), FEV1/FVC, and vital capacity (VC) were measured using a computerized spirometer (Spiro lab 3). The intrarater reliability for upper and lower CE showed *very good agreement* with intraclass correlation (ICC) values between 0.90 and 0.93 for upper CE and 0.85 to 0.86 for lower CE. The interrater reliability for upper CE showed good to *very good agreement* with ICC values ranging between 0.78 and 0.83, and lower CE showed *very good agreement* with ICC values ranging between 0.82 and 0.84. Upper and lower CE showed a significant and positive correlation with all lung function parameters, with strong correlation with FEV1/FVC (*r* = 0.68). Upper and lower CE measurements with inch tape showed good intra- and interrater reliability and reproducibility in healthy nonsmokers, healthy smokers, and COPD subjects. Compared to upper, lower CE correlated well with the lung function parameters. Upper and lower CE may be more useful in clinical practice to evaluate chest mobility and to give indirect information on lung function but interpretation with caution is required when considering implementation into clinical setting.

## 1. Introduction

Noninvasive methods of monitoring respiratory function have gained increasing interest recently, particularly measures of chest wall movement [1]. The chest wall distortion measurement allows objective assessment of the synchronous and asynchronous behavior of the rib cage during breathing. Subjects with respiratory dysfunction may exhibit alterations in chest wall mobility resulting in chest wall stiffness and abnormal chest biomechanics [2]. In diseases such as asthma and chronic obstructive pulmonary disease (COPD), rib cage mobility may be decreased as a result of hyperinflation, airway obstruction, and mechanical disadvantage of the respiratory muscles [3]. Smoking is associated with major health risks affecting both respiratory and cardiovascular systems [4]. Inhaling cigarette smoke causes alterations in airflow resistance and irritation in the airways resulting in alterations in respiratory function [5]. Respiratory function evaluation tests like chest expansion (CE) may indicate deterioration in respiratory function prior to the commencement of clinical symptoms.

Evaluation of chest wall mobility is considered the most important tool for assessing abnormal respiratory patterns at rest and during exercise. Noninvasive methods are required to determine respiratory patterns, as invasive methods affect respiratory movement patterns [6]. Respiratory plethysmography via induction, magnetometry, and optoelectronic plethysmography are noninvasive methods that have been considered in the literature and found to meet acceptable standards but are expensive and require trained technicians [6–9]. Studies have used these methods to evaluate pre- and postintervention changes without previously testing the reliability of interventions [10, 11]. Therefore, inexpensive, simple, accurate, and reproducible evaluation methods and tools are required to assess chest wall mobility.

Measurement of CE was first described by Moll et al. in 1972 [12]. CE measurement has been used in different disease conditions to assess the effects of different treatment techniques, including respiratory muscle endurance training and respiratory muscle stretching [13, 14]. CE is commonly measured as the difference between the thoracic girth measurement after maximal inspiration and at the end of maximal expiration [1]. Different anatomical reference markers and different arm positions have been used for measures of CE, and this may have contributed to differences in analysis and interpretation [15–17]. Anatomical reference markers for upper CE include the fourth intercostal space, axillary line, and 5th thoracic vertebrae, and lower CE include xiphoid process and 10th thoracic vertebrae [1, 18–20].

CE seems to be diverse and variable within healthy and diseased subjects, ranging from 4–7 cm in healthy subjects [16, 21]. The normal range of CE decreases with age (50 to 60% between 15 y and 75 y) and 20% more in men compared to women [12]. Although published studies have regularly used the CE measurement, investigation of its properties and standardization of the procedure is lacking and performed results have been conflicting [16, 17]. Pile et al. showed poor coefficient of reliability of CE as 0.15 in 10 ankylosing spondylitis (AS) subjects [17]. Malaguti et al. showed fair to good reliability (ICC 0.69 to 0.89) by two examiners making the measurements on the same day in subjects with COPD [22]. Sharma et al. showed very good intraclass correlation coefficients (ICC) (intratester reliability as 0.85 to 0.97, and intertester reliability as 0.93 to 0.97) in 22 AS and 25 healthy subjects [16]. These performed study results have been conflicting, and there are limited studies that consider the upper and lower CE reliability in diverse population with standardized procedure. Therefore, the objective of this study is to evaluate intrarater and interrater reliability of CE measurements taken at two different levels of chest positions (upper and lower).

Previous studies were conducted to see the relationship between CE and pulmonary function [19, 23]. Different authors reported a considerable decrease in chest wall mobility with aging and chronic chest diseases [1, 19, 24]. Upper and lower CE may be more useful in clinical practice to evaluate chest mobility and to give indirect information on lung function. Although associations between chest wall mobility, lung function, have been reported in patients with ankylosing spondylitis and fibromyalgia, there is a paucity of studies on chest wall mobility in healthy smokers and COPD subjects [19, 25]. Therefore, this study investigates the correlation between CE measurements and lung function measures i.e., forced expiratory volume in first second (FEV1), forced vital capacity (FVC), and FEV1/FVC and vital capacity (VC) obtained from spirometry in healthy nonsmokers, healthy smokers, and patients with COPD.

## 2. Materials and Methods

### 2.1. Subjects

Healthy nonsmokers and healthy smokers were recruited for this cross-sectional study from King Khalid University, Saudi Arabia. COPD patients with mild, moderate, and severe symptoms were recruited from government hospitals of the local town (study duration: January to August 2017). Healthy nonsmokers are subjects that have never attempted smoking cigarettes. Healthy smokers are regular smokers that smoke at least 15 cigarettes per day. Other inclusion criteria include absence of musculoskeletal disorders, respiratory and neuromuscular disease, and no other factors present that might alter respiratory biomechanics. Inclusion criteria for COPD patients include stable with no recent changes in medication, not requiring supplemental oxygen, not taking oral corticosteroids, and no exacerbations present for the preceding 5 weeks. Patients were excluded if any comorbidities were present, such as heart disease, bronchial asthma, bronchiectasis, pulmonary fibrosis, ankylosing spondylitis, and any chest wall deformities. The university ethics committee approved this study (REC # 2016-08-06), and all subjects who met the inclusion criteria read the instructions and signed a written informed consent prior to commencement of the study.

### 2.2. Chest Expansion Measurement

**Figure 1:**
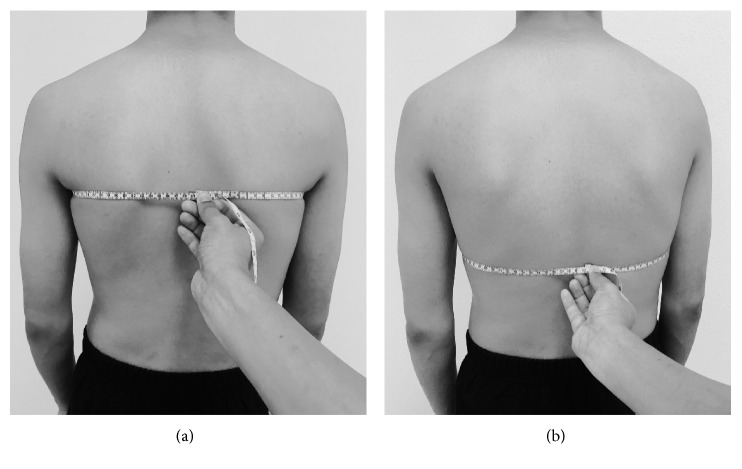
Measurement procedure of (a) upper chest expansion and (b) lower CE.

A measuring tape was used to measure CE in centimeters (cm) at two levels of the rib cage. For upper CE (Figure 1(a)), the anatomical landmarks used were the spinous process of fifth thoracic vertebrae, the middle of the clavicular line, and the third intercostal space [26]. For lower CE (Figure 1(b)), the anatomical landmarks used were the spinous process of 10th thoracic vertebrae and the xiphoid process [26]. Each therapist was alone with the patient and was blinded to the other measures of the therapist. The order of testing CE by the two examiners were randomized using flipping a coin. The side of the coin (i.e., heads: examiner A, tails: examiner B) determines the assignment to each subject. The examiner testing the subject initially evaluated CE measurements first on day one and two.

### 2.3. Instructions to Subjects

The instructions given by the therapist to subjects during breathing was standardized. Prior to the thoracic measurements, subjects were asked to “inhale slowly and rhythmically through the nose against the inch tape to open up the lungs as much as you can,” and then the subjects were asked to “exhale through the mouth completely.” CE measurement was taken at the end of the inspiration and expiration cycles, while the subject was in a standing position with their arms at the side of their body. The examiner placed the “0” point of the measuring tape (starting tip) on the spinous process of the vertebrae. The tape was secured by the index finger of the examiner between the subject's body and the tape without generating extra pressure (Figure 1). To calculate the CE value, the inspiratory diameter was subtracted from the expiratory diameter. Intrarater (same day) and interrater (between day) reliabilities were evaluated by repeated measurement by both examiners on one or two separate days. On each occasion, three trials of CE measurement procedure were recoded, and the average was measurement was used for analysis.

### 2.4. Lung Function Measurement

A computerized spirometer (Spiro lab 3; Medical International Research MIR, Italy) with a standard mouthpiece was used to measure the lung function following the guidelines of the American Thoracic Society (ATS). This computerized spirometer conducts the breathing tests and calculates an index of test quality and control. FVC, FEV1, FEV1/FVC, VC, and IC measurements are made with subjects in a sitting position. The computer then gives a functional interpretation with 11 possible levels following the ATS and European Respiratory Society (ERS) classification [27].

Prior to commencement of the testing, all subjects were familiarized with the test procedures and were allowed to do multiple trials prior to the testing. While performing spirometry, a mouthpiece made of cardboard without teeth grip was used, and the subject held the mouthpiece tightly with the nose closed with the nose clip. All subjects completed a minimum of three trials with the best (highest) test result kept for analysis. A minimum 3-minute rest was given between each trial. All the subjects were given the same instructions while performing the tests to avoid bias.

### 2.5. Statistical Analysis

Statistical analyses were performed using SPSS 20.0 (IBM-SPSS Inc., Armonk, NY). The data were expressed as mean ± SD. One-way ANOVA was performed to see baseline characteristics and CE differences among three groups. Turkey Post hoc analysis was performed to know the respective difference of each group compared to the others. Intrarater and interrater reliability were assessed using intraclass correlation (ICC) agreement values (two-way mixed effects model, consistency definition) with 95% confidence interval (CI). For evaluating agreement between rater scores, Bland–Altman's limits of agreements (LOA) was used [28]. Furthermore, measurement errors were estimated by calculating the standard error of measurement (SEM) using the following formula: SEM consistency = SD difference/√2 (SD difference = standard deviation of the mean differences between examiners A and B). The smallest detectable change (SDC) was calculated using the following formula: 1.96 × √2 × SEM [29, 30]. We interpreted ICC agreement values as follows: >0.80 was very good, 0.61–0.80 was good, 0.41–0.60 was moderate, 0.21–0.40 was fair, and <0.21 was poor [31]. Adequate sample size is required to achieve an admissible 95% CI for ICC values, and a sample size of 50 participants is recommended to assess reliability [22, 32]. Pearson coefficients were calculated to assess correlations between chest expansions (upper and lower) and lung function parameters assessed on first day. The significance level was set at *p* < 0.05 for all the tests.

## 3. Results

Seventy subjects (25 healthy nonsmokers, 25 healthy smokers, and 20 COPD) aged between 18 and 70 years participated in the study. Anthropometric characteristics and lung function data for the study population are summarized in Table 1. There was a significant difference in baseline demographic and lung parameter characteristics between groups (*p* ≤ 0.001). Post hoc analysis between groups showed statistically respective difference (*P* < 0.05) of each group compared to the others except for age, BMI, FVC, FEV1/FVC, and VC (*p* > 0.05) between healthy nonsmokers and healthy smokers groups.

The mean upper CE in healthy nonsmokers ranged from 5.6 to 6.4 cm, in healthy smokers it ranged from 5.4 to 5.9 cm, and COPD subjects ranged from 3.7 to 4.4 cm (Table 2). The mean lower CE in healthy nonsmokers ranged from 7.0 to 7.5 cm, healthy smokers ranged from 7.5 to 8.0 cm, and COPD subjects ranged from 4.8 to 5.2 cm. There was a significant difference (*p* ≤ 0.001) in upper and lower CE measurements between the three groups (Table 2).

### 3.1. Intrarater Reliability

**Figure 2:**
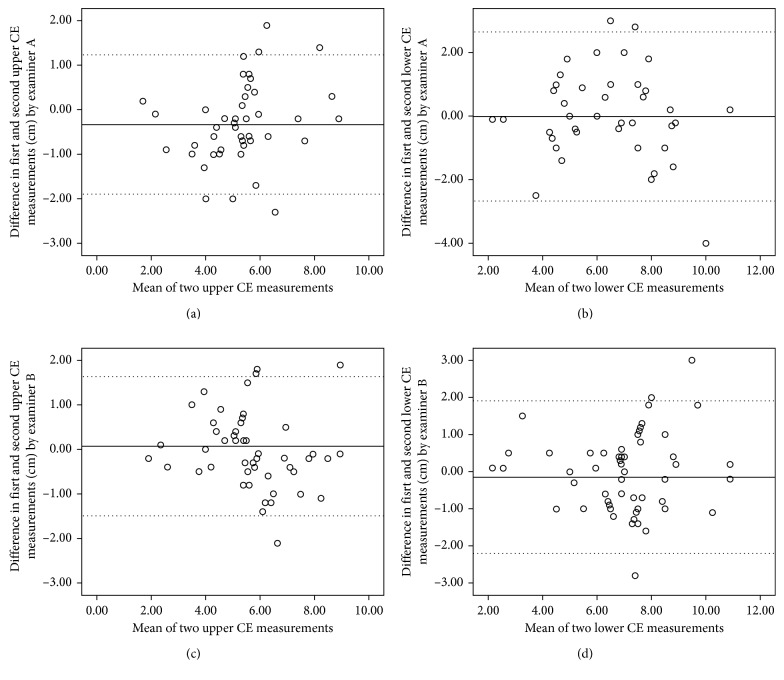
Bland–Altman plots of intrarater reliability for upper (a, c) and lower (b, d) CE measurements by examiners A and B. The solid lines indicate the reference mean. The dotted lines indicate the upper and lower limits of agreement.

The intrarater reliability for upper and lower CE showed *very good agreement,* with ICC values of 0.90 and 0.93 for upper CE, and of 0.85 and 0.86 for lower CE (Table 3). The Bland–Altman plots with mean and LOA for both examiners are shown in Figure 2. Examiner B had higher ICC values for upper CE (0.93 (95% CI [0.89–0.95])) with 95% LOA of −0.64 to 0.95 cm. The SDC ranged from 2.24 to 3.6 cm and SEM ranged from 0.81 to 1.30 cm (Table 3). Examiner A had higher ICC values for lower CE (0.86 (95% CI [0.78–0.91])) with a 95% LOA of 2.65–2.67 cm. The SDC ranged from 2.24 cm to 3.6 cm, and SEM ranged between 3.90 cm and 4.40 cm (Table 3).

### 3.2. Interrater Reliability

**Figure 3:**
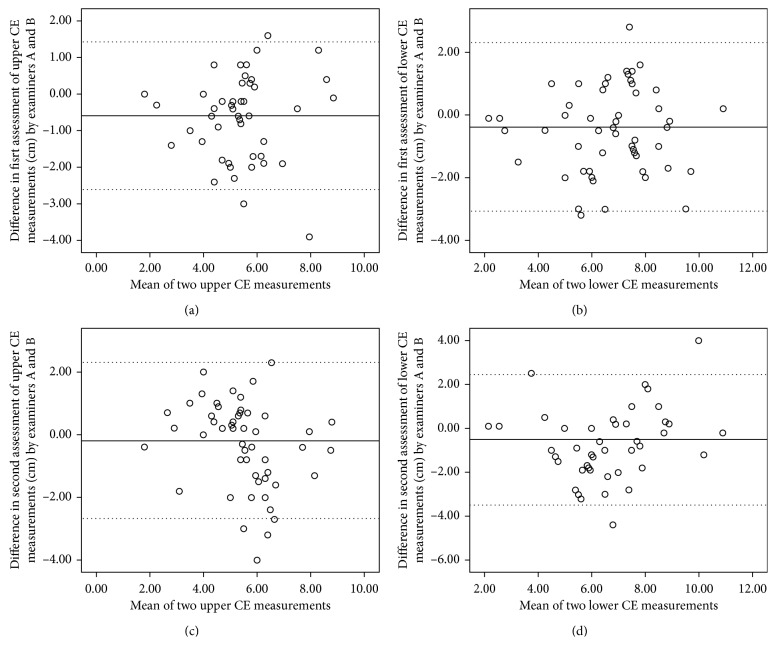
Bland–Altman plots of interrater reliability for upper (a, c) and lower (b, d) CE measurements by examiners A and B. The solid lines indicate the reference mean. The dotted lines indicate the upper and lower limits of agreement.

Overall, the interrater reliability for upper CE showed *good to very good agreement* with ICC values of 0.78 and 0.83 and lower CE showed *very good agreement* with ICC values of 0.82 and 0.84 (Table 3). The Bland–Altman plots with mean and LOA for first and second assessments are presented in Figure 3. For upper and lower CEs, the first assessment demonstrated very *good agreement* with an ICC value of 0.83 (95% CI [0.64–0.91]) for upper CE and with an ICC value of 0.84 (95% CI [0.74–0.90]) for lower CE (Table 3 and Figure 3). Both CEs showed *good* to *very good agreement* with ICC ≥0.78. SDCs were between 2.24 and 4.40 cm (Table 3). Overall, the mean differences between the examiners ranged between −0.18 cm (SD = 1.27) and −0.59 cm (SD = 1.03) (Table 3). The overall SDCs for upper CE were between 2.24 and 3.60 and lower CE were 3.90 to 4.40.

### 3.3. Chest Expansion Correlations with Lung Function Parameters

**Figure 4:**
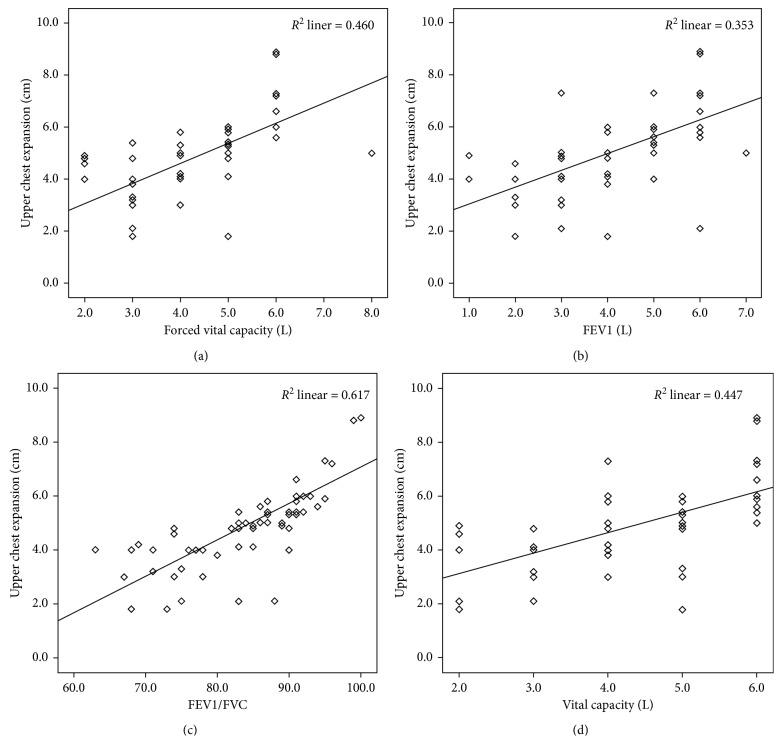
Relationship between upper CE measurement (cm) and (a) forced vital capacity (FVC), (b) forced expiratory volume in first second (FEV1), (c) FEV1/FVC, and (d) vital capacity (VC).

**Figure 5:**
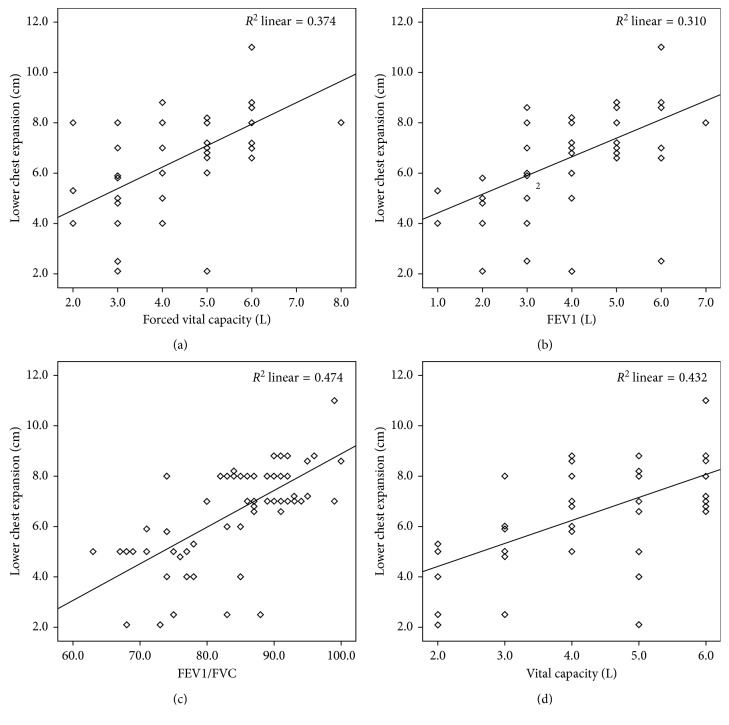
Relationship between lower CE measurement (cm) and (a) forced vital capacity (FVC), (b) forced expiratory volume in first second (FEV1), (c) FEV1/FVC, and (d) vital capacity (VC).

Pearson correlation coefficients indicate positive correlations between CE values and lung function parameters (Figures 4 and 5). Upper CE showed moderate (IC [*r* = 0.40]) to strong (FEV1/FVC [*r* = 0.78]) correlations; all lung function measures were significantly correlated to upper CE with *p* < 0.001 (Table 4). Lower CE also showed moderate (IC [*r* = 0.46]) to strong (FEV1/FVC [*r* = 0.68]) correlations, and again, all lung function correlations with lower CE were statistically significant at *p* < 0.001 (Table 4).

## 4. Discussion

To our knowledge, this study is first to demonstrate the reliability of chest wall measurements in healthy nonsmokers, healthy smokers, and COPD tested together and to confirm that chest wall measurement correlates with lung function. ICC showed that intra- and interrater reliability of upper and lower CE measurements was *good to very good* and that both upper and lower CE measurements are significantly, positively correlated with lung function parameters.

Reliability studies are necessary to determine the variability of an assessment method. Knowing the variability of an assessment method is crucial to prevent interpretation errors when using an assessment to compare measures pre- and posttreatment (or other interventions). Previous studies have assessed the reliability of frequently performed tests and assessments in clinical practice and rehabilitation setups, such as walking tests, spirometry, quality of life, and others [33]. Thoracic chest measurement is another commonly measured pre- and postintervention metric in subjects with cardiorespiratory problems [10]. However, the reliability of chest wall measurements tested in various populations within a single study was not previously available.

In this study, a wide range of upper and lower CE values was observed among subjects, indicating that there is variability in the measure. The same variability has been noted in previously published studies [12, 15]. Mean CE measurement values were greater in healthy nonsmokers and healthy smokers when compared with previous studies published. This could be due to the younger age of participants in this study [12]. Debouche and colleagues, who also included young healthy subjects in their study, showed similar upper and lower CE measurement ranges (upper CE: 5.4–5.7 cm; lower CE: 6.4–6.8 cm) as ours (upper CE: 5.4–5.9 cm; lower CE: 7.0–8 cm) [26]. In agreement with other previously published studies, this study shows that lower CE measurements are larger than upper CE values in healthy nonsmokers, healthy smokers, and COPD subjects. In contrast, a study by Malaguti and colleagues found that upper CE was larger than lower CE in subjects with COPD [22].

Upper and lower CE measurements in this study showed good intra- and interrater reliability. Previously published research on upper and lower levels of CE measurements have found good to very good intra- and interreliability, ranging from 0.69 to 0 0.93 and 0.64 to 0.95, respectively, with results statistically significant [15, 16, 18]. Our study results show similar ICCs to these previous studies. Although the ICCs are similar, the results cannot be compared, as the study methodological considerations are different from this study in terms of study population, subject position, and anatomical markers for CE measurement.

This study included subjects that were healthy nonsmokers, healthy smokers, and COPD; all were measured by two therapists to assess the coefficient of variability for CE, and good reproducibility was seen for both upper and lower CE measurements. The mean differences between therapists were 0.33 and 0.80 cm for upper CE, and 0.01 and 0.05 cm for lower CE. 0.18 cm and 0.59 cm between days. The mean between day differences of first and second assessment of upper and lower CE of examiner A was 0.59 cm and 0.38 cm and of examiner B was 0.18 cm and 0.52 cm. These small differences are within acceptable limits and are not statistically or clinically significant.

Monitoring the effects of treatment is of well-recognized importance and is the foundation of modern evidence-based health care. SDC and minimal clinically important difference (MCID) can be used as benchmarks for the interpretability of a CE to determine whether the observed change is beneficial to the patients. To determine whether a change score on an individual patient level is clinically important and not just measurement error, the SDC score must not exceed the MCID change score [4]. In this study, SDCs for upper CE were between 2.24 and 3.60, and for lower CE, they were 3.90 to 4.40. Further studies considering CE measurement as an outcome measure, the MCID change scores should be above 3.60 for upper CE and 4.40 for lower CE. Though CE is a useful measure, its usefulness for repeated measures in individual patients may not be justified by the results of this study. The SDC ranges for upper and lower CE in this study are high. However, the volume of changes required in CE should be large enough to see a real change. Therefore, interpretation with caution is required when considering implementation into the clinical practice.

The results of this study shows upper and lower CE measurements significantly correlate with lung function parameters (FVC, FEV1, FEV1/FVC, and VC). The strongest correlation was between CE and FEV1/FVC (*r* = 0.68 and 0.78 for lower and upper CE, respectively). These findings may be favored due to greater compliance of chest wall in standing position preferred in this study. A positive correlation between CE and VC has previously been found in healthy [20] and ankylosing spondylosis subjects [19]. Debouche et al. found a significant correlation between CE and all lung function parameters (FEV1, FVC, VC, and maximal inspiratory pressure (MIP), except FEV1/FVC). [26] In contrast to our study, Malaguti et al. found no correlation between CE and pulmonary function parameters in subjects with COPD [22]. In spite of the pathology involved in subjects with COPD (hyperinflation and low diaphragm), this study showed correlations between CE and lung function parameters. These results may have been different if only the COPD subjects alone are compared for correlation between CE and lung function parameters. In this study, upper CE showed a stronger correlation with all lung function parameters compared to lower CE. In contrast, Debouche et al. found that lower CE had a stronger correlation with lung function parameters [26]. These differences may be due to the fact that only healthy subjects were included in the study of Debouche et al., whereas this study included both healthy and pathology subjects. A significant positive correlation between CE and maximal inspiratory pressure has been observed in subjects who were healthy or had osteoporosis or fibromyalgia [34, 35]. These similar findings and relationships are observed in this study. This study demonstrates intra- and interrater reliability in three types of subjects. The sample size of COPD subjects included in this study was smaller than that of the other two populations. Results might be different if the COPD sample is increased. This study also shows chest wall mobility is closely associated with FVC and FEV1/FVC, and this finding is in accordance with previous study in subjects with ankylosing spondylitis [19, 36]. Therefore, maintaining chest wall mobility may be an important element for preserving FVC and FEV1/FVC in elderly male patients with COPD.

This study used a standing position for measuring a subject's upper and lower CE, as it is a similar position as those chosen in several previous studies although different positions have also been used [1, 15, 21, 37]. The standing position was favored as the manipulation of the measuring tape was easier in this position. This position also improves thoracic over abdominal breathing, and this difference is important when the subjects take air into the lungs and then expel it with larger volumes [24]. Some authors have measured CE with hands on the head to prevent shoulder adduction, which facilitates CE, and authors of that study argue that manipulation of the tape and reading is easy in this position [12]. This study measured CE with subjects keeping the hands alongside the body, and subjects accepted this position as the most comfortable [16]. This position does not influence measurement and reproducibility [16]. Further, subjects with shoulder stiffness or dysfunction might have difficulty placing the hands on the head.

### 4.1. Limitations of the Study

In this study, all the subjects participated were male; the external validity may be compromised, considering the respiratory patterns among sex. In the present study, the ICC for intrarater and intertester reliability appears higher. This is, in part, possibly due to the method of calculation. The average from six trials of CE score on two occasions for each tester was used to calculate the reliability. According to Portney and Watkins, the ICC based on mean rating always shows higher reliability than one based on single ratings [38]. We demonstrated the intra- and interrater reliability using large group of healthy nonsmokers, healthy smokers and small group COPD subjects. However, the results of our study should be confirmed in patients when thoracic compliance is impaired by processes affecting the respiratory pump, such as neuromuscular or chest wall diseases. Moreover, to be complete, responsiveness should be evaluated on a large sample of patients for various interventions and a minimal clinically important difference for these tools should be determined. The examiners participated in the study had good expertise in the field of physical therapy and in evaluating CE measurement, so the intra- and interrater reliability was found good. “If” the data were to be collected with novice physical therapists, then the reliability values may change.

## 5. Conclusion

Upper and lower CE measurements taken with a measuring tape have good intra- and interrater reliability and reproducibility in healthy nonsmokers, healthy smokers, and COPD subjects. The reliability found in the present study can be credited to anatomical landmarks selected with standardized procedure. Though reliability is good, the usefulness of CE and its usefulness for interpreting disease progression and efficacy of intervention needs caution. Upper and lower CE measures correlated with lung function parameters measured by spirometer (FVC, FEV1, FEV1/FVC, and VC). Upper and lower CE may be more useful in clinical practice to evaluate chest mobility and to give indirect information on lung function, but interpretation with caution is required when considering implementation into clinical setting.

## Figures and Tables

**Table 1:** Demographic and lung parameter characteristics of healthy nonsmokers, healthy smokers, and COPD patients.

	Healthy nonsmokers (*n*=25) Mean ± SD	Healthy smokers (*n*=25) Mean ± SD	COPD patients (*n*=20) Mean ± SD	*P* value for group difference
Age (years)	23.6 ± 5.3	23.6 ± 3.8	52.0 ± 13.7	<0.001
BMI (kg/m^2^)	24.1 ± 3.8	23.8 ± 4.9	29.5 ± 6.7	0.001
FEV1 (L)	5.1 ± 0.6	4.2 ± 0.9	2.5 ± 0.8	<0.001
FVC (L)	5.1 ± 0.8	5.2 ± 1.0	3.0 ± 0.7	<0.001
FEV1/FVC	90.7 ± 3.6	88.0 ± 3.5	73.7 ± 5.6	<0.001
VC (L)	5.2 ± 1.0	4.8 ± 0.9	3.1 ± 1.0	<0.001

COPD = chronic obstructive pulmonary disease; BMI = body mass index; FEV1 = forced expiratory volume in 1 second; FVC = forced vital capacity. Values are mean ± SD unless otherwise indicated.

**Table 2:** Chest expansion values and difference between the groups.

		Examiner A, first assessment		Examiner A, second assessment		Examiner B, first assessment		Examiner B, second assessment	
		Upper CE (cm)	Lower CE (cm)	Upper CE (cm)	Lower CE (cm)	Upper CE (cm)	Lower CE (cm)	Upper CE (cm)	Lower CE (cm)
Healthy nonsmokers	Mean	5.6	7.0	5.6	7.2	6.1	7.2	6.4	7.5
	SD	1.8	2.0	1.5	1.8	1.7	1.9	1.7	2.0
	Variance	3.4	2.0	2.4	2.5	3.1	2.9	3.0	2.3
	Minimum	2.1	2.5	2.2	2.6	2.4	2.6	2.3	2.5
	Maximum	8.9	11	9.0	10.8	9.9	10.8	9.0	11.0

Healthy smokers	Mean	5.4	7.7	5.9	7.5	5.9	7.6	5.5	8.0
	SD	0.8	2.6	0.8	1.6	0.8	1.5	1.0	1.2
	Variance	0.7	2.0	0.7	2.6	0.7	2.3	1.0	1.6
	Minimum	4.1	6.0	5.0	5.0	5.0	5.0	4.1	6.0
	Maximum	7.3	10.3	8.0	11.0	7.7	11.0	7.9	10.8

COPD	Mean	3.7	4.9	4.2	4.8	4.3	5.2	4.4	4.7
	SD	0.8	1.4	1.0	1.8	1.3	2.2	1.6	2.6
	Variance	0.7	2.1	1.1	2.4	1.7	2.5	2.7	1.8
	Minimum	1.8	2.1	1.6	2.2	1.8	2.2	2.0	2.1
	Maximum	4.9	8.0	5.8	7.8	6.2	8.6	6.3	7.4

*p* value		<0.001	<0.001	<0.001	<0.001	0.001	<0.001	0.040	0.011

**Table 3:** Intrarater and interrater reliability for upper and lower chest expansions.

	ICC agreement	95% CI	Mean diff._AB (SD)	SEM consistency	LOA	SDC
*Examiner A*						
Upper chest expansion	0.90	0.83–0.94	−0.33 (0.80)	0.88	1.23–1.89	2.43
Lower chest expansion	0.86	0.78–0.91	−0.01 (1.36)	1.41	2.65–2.67	3.90
*Examiner B*						
Upper chest expansion	0.93	0.89–0.95	0.80 (0.08)	0.81	−0.64–0.95	2.24
Lower chest expansion	0.85	0.77–0.90	−0.15 (1.05)	1.45	1.90–2.20	4.01
*First assessment*						
Upper chest expansion	0.83	0.64–0.91	−0.59 (1.03)	1.18	1.42–2.60	3.27
Lower chest expansion	0.84	0.74–0.90	−0.38 (1.37)	1.42	2.30–3.06	3.93
*Second assessment*						
Upper chest expansion	0.78	0.66–0.86	−0.18 (1.27)	1.30	2.30–2.66	3.60
Lower chest expansion	0.82	0.70–0.89	−0.52 (1.52)	1.59	2.45–3.49	4.40

95% CI = 95% confidence interval; ICC agreement = intraclass correlation coefficients. Mean diff_AB = mean difference between examiner A and B; SEM = standard error of measurement; LOA = limits of agreements; SDC = smallest detectable change.

**Table 4:** Coefficient of correlation between chest expansions and lung function parameters.

		FVC	FEV1	FEV1/FVC	VC
Upper CE	r	0.678	0.595	0.785	0.668
	p	<0.001	<0.001	<0.001	<0.001

Lower CE	r	0.611	0.557	0.689	0.658
	p	<0.001	<0.001	<0.001	<0.001

CE = chest expansion; VC = vital capacity; FVC = forced vital capacity; FEV1 = forced expiratory volume in 1 second (this table is reproduced from Debouche et al. [24], under creative commons attribution license/public domain).

## Data Availability

The data used to support the findings of this study are included within the supplementary information files.

## Supplementary Materials

Supplementary Materials The Supplementary Materials file is the SPSS data set used to support the findings of this study.

## References

[1] Moll J. M., Wright V. (1972). An objective clinical study of chest expansion. *Annals of the Rheumatic Diseases*.

[2] Guerin C., Coussa M. L., Eissa N. T. (1993). Lung and chest wall mechanics in mechanically ventilated COPD patients. *Journal of Applied Physiology*.

[3] Jubran A., Tobin M. J. (1992). The effect of hyperinflation on rib cage-abdominal motion. *American Review of Respiratory Disease*.

[4] Terwee C. B., Roorda L. D., Knol D. L., De Boer M. R., De Vet H. C. W. (2009). Linking measurement error to minimal important change of patient-reported outcomes. *Journal of Clinical Epidemiology*.

[5] Thomson N. C., Chaudhuri R., Livingston E. (2004). Asthma and cigarette smoking. *European Respiratory Journal*.

[6] Cohn M., Rao A., Broudy M. (1982). The respiratory inductive plethysmograph: a new non-invasive monitor of respiration. *Bulletin Europeen de Physiopathologie Respiratoire*.

[7] Fernandes M., Cukier A., Ambrosino N., Leite J., Feltrim M. Z. (2016). Respiratory pattern, thoracoabdominal motion and ventilation in chronic airway obstruction. *Monaldi Archives for Chest Disease*.

[8] Fitting J., Grassino A. (1986). Technics for the functional evaluation of the thoracic cage. *Revue des Maladies Respiratoires*.

[9] Aliverti A., Stevenson N., Dellaca R., Mauro A. L., Pedotti A., Calverley P. (2004). Regional chest wall volumes during exercise in chronic obstructive pulmonary disease. *Thorax*.

[10] Putt M. T., Watson M., Seale H., Paratz J. D. (2008). Muscle stretching technique increases vital capacity and range of motion in patients with chronic obstructive pulmonary disease. *Archives of Physical Medicine and Rehabilitation*.

[11] Minoguchi H., Shibuya M., Miyagawa T. (2002). Cross-over comparison between respiratory muscle stretch gymnastics and inspiratory muscle training. *Internal Medicine*.

[12] Moll J. M. H., Liyanage S. P., Wright A. V. (1972). An objective clinical method to measure lateral spinal flexion. *Rheumatology*.

[13] Lemaitre F., Coquart J. B., Chavallard F (2013). Effect of additional respiratory muscle endurance training in young well-trained swimmers. *Journal of Sports Science and Medicine*.

[14] Johansson E.-L., Ternesten-Hasséus E., Olsén M. F., Millqvist E. (2012). Respiratory movement and pain thresholds in airway environmental sensitivity, asthma and COPD. *Respiratory Medicine*.

[15] Bockenhauer S. E., Chen H., Julliard K. N., Weedon J. (2007). Measuring thoracic excursion: reliability of the cloth tape measure technique. *Journal of the American Osteopathic Association*.

[16] Sharma J., Senjyu H., Williams L., White C. (2004). Intra-tester and inter-tester reliability of chest expansion measurement in clients with ankylosing spondylitis and healthy individuals. *Journal of the Japanese Physical Therapy Association*.

[17] Pile K. D., Laurent M. R., Salmond C. E., Best M. J., Pyle E. A., Moloney R. O. (1991). Clinical assessment of ankylosing spondylitis: a study of observer variation in spinal measurements. *Rheumatology*.

[18] Rahali-Khachlouf H., Poiraudeau S., Fermanian J., Ben F. S., Dziri C., Revel M. (2001). Validity and reliability of spinal clinical measures in ankylosing spondylitis. *Annales de Readaptation et de Medecine Physique: Revue Scientifique de la Societe Francaise de Reeducation Fonctionnelle de Readaptation et de Medecine Physique*.

[19] Fisher L. R., Cawley M. I., Holgate S. T. (1990). Relation between chest expansion, pulmonary function, and exercise tolerance in patients with ankylosing spondylitis. *Annals of the Rheumatic Diseases*.

[20] de Cordoba Lanza F., de Camargo A., Archija L. R. F., Selman J. P. R., Malaguti C., Dal Corso S. (2013). Chest wall mobility is related to respiratory muscle strength and lung volumes in healthy subjects. *Respiratory Care*.

[21] Gouilly P., Reggiori B., Gnos P. L., Schuh O., Muller K., Dominguez A. (2009). À propos de la mesure de l’ampliation thoracique. *Kinésithérapie, la Revue*.

[22] Malaguti C., Rondelli R. R., de Souza L. M., Domingues M., Dal Corso S. (2009). Reliability of chest wall mobility and its correlation with pulmonary function in patients with chronic obstructive pulmonary disease. *Respiratory Care*.

[23] Chanavirut R., Khaidjapho K., Jaree P., Pongnaratorn P. (2006). Yoga exercise increases chest wall expansion and lung volumes in young healthy Thais. *Physiology (The American Physiological Society)*.

[24] Verschakelen J. A., Demedts M. G. (1995). Normal thoracoabdominal motions. Influence of sex, age, posture, and breath size. *American Journal of Respiratory and Critical Care Medicine*.

[25] Ozgocmen S., Cimen O. B., Ardicoglu O. (2014). Relationship between chest expansion and respiratory muscle strength in patients with primary fibromyalgia. *Clinical Rheumatology*.

[26] Debouche S., Pitance L., Robert A., Liistro G., Reychler G. (2016). Reliability and reproducibility of chest wall expansion measurement in young healthy adults. *Journal of Manipulative and Physiological Therapeutics*.

[27] Tammeling G., Cotes J., Pedersen O., Peslin R., Yernault J. (1993). Standardized lung function testing. *European Respiratory Journal*.

[28] Bland J. M., Altman D. (1986). Statistical methods for assessing agreement between two methods of clinical measurement. *The Lancet*.

[29] de Vet H. C. W., Terwee C. B., Knol D. L., Bouter L. M. (2006). When to use agreement versus reliability measures. *Journal of Clinical Epidemiology*.

[30] Mokkink L. B., Terwee C. B., Patrick D. L. (2010). The COSMIN checklist for assessing the methodological quality of studies on measurement properties of health status measurement instruments: an international Delphi study. *Quality of Life Research*.

[31] Altman D. G. (1990). *Practical Statistics for Medical Research*.

[32] Terwee C. B., Bot S. D. M., de Boer M. R. (2007). Quality criteria were proposed for measurement properties of health status questionnaires. *Journal of Clinical Epidemiology*.

[33] Romer L. M., McConnell A. K. (2004). Inter-test reliability for non-invasive measures of respiratory muscle function in healthy humans. *European Journal of Applied Physiology*.

[34] Cimen O. B., Ulubas B., Sahin G., Calikoglu M., Bagis S., Erdogan C. (2003). Pulmonary function tests, respiratory muscle strength, and endurance of patients with osteoporosis. *Southern Medical Journal*.

[35] Ozgocmen S., Ardicoglu O. (1999). Reduced chest expansion in primary fibromyalgia syndrome. *Yonsei Medical Journal*.

[36] Elliott C., Hill T., Adams T., Crapo R., Nietrzeba R., Gardner R. (1985). Exercise performance of subjects with ankylosing spondylitis and limited chest expansion. *Bulletin Europeen de Physiopathologie Respiratoire*.

[37] Caldeira V. d. S., Starling C. C. D., Britto R. R., Martins J. A., Sampaio R. F., Parreira V. F. (2007). Precisão e acurácia da cirtometria em adultos saudáveis. *Jornal Brasileiro de Pneumologia*.

[38] Portney L., Watkins M. (1993). *Foundations of Clinical Research: Application to Practice*.

